# The electrostatic profile of consecutive Cβ atoms applied to protein structure quality assessment

**DOI:** 10.12688/f1000research.2-243.v3

**Published:** 2014-09-16

**Authors:** Sandeep Chakraborty, Ravindra Venkatramani, Basuthkar J. Rao, Bjarni Asgeirsson, Abhaya M. Dandekar

**Affiliations:** 1 Department of Biological Sciences, Tata Institute of Fundamental Research, Mumbai, 400 005, India; 2 Department of Chemical Sciences, Tata Institute of Fundamental Research, Mumbai, 400 005, India; 3 Science Institute, Department of Biochemistry, University of Iceland, IS-107 Reykjavik, Iceland; 4 Plant Sciences Department, University of California,, Davis, CA, 95616, USA

## Abstract

The structure of a protein provides insight into its physiological interactions with other components of the cellular soup. Methods that predict putative structures from sequences typically yield multiple, closely-ranked possibilities. A critical component in the process is the model quality assessing program (MQAP), which selects the best candidate from this pool of structures. Here, we present a novel MQAP based on the physical properties of sidechain atoms. We propose a method for assessing the quality of protein structures based on the electrostatic potential difference (EPD) of Cβ atoms in consecutive residues. We demonstrate that the EPDs of Cβ atoms on consecutive residues provide unique signatures of the amino acid types. The EPD of Cβ atoms are learnt from a set of 1000 non-homologous protein structures with a resolution cuto of 1.6 Å obtained from the PISCES database. Based on the Boltzmann hypothesis that lower energy conformations are proportionately sampled more, and on Annsen's thermodynamic hypothesis that the native structure of a protein is the minimum free energy state, we hypothesize that the deviation of observed EPD values from the mean values obtained in the learning phase is minimized in the native structure. We achieved an average specificity of 0.91, 0.94 and 0.93 on hg_structal, 4state_reduced and ig_structal decoy sets, respectively, taken from the Decoys `R' Us database. The source code and manual is made available at https://github.com/sanchak/mqap and permanently available on 10.5281/zenodo.7134.

## Introduction

The challenge of deriving the native structure of a protein from its sequence has intrigued researchers for decades ^1^. Methods that predict putative structures from sequences are based either on features from databases of known structures (template-based methods) ^2– 4^ or use first principles of atomic interactions ( *ab initio* or *de novo* methods) ^5– 7^. Typically, these methods yield multiple, closely-ranked possibilities. Model quality assessment programs (MQAP) that validate accuracy of these predicted structures are used to select the best candidate from the set of predicted structures.

MQAPs can be classified as energy, consensus or knowledge based. Two major sources of errors in energy based methods used for refining or discriminating protein structures are inaccuracies in the force field due to the inherent approximations in equations that model multi-atomic configurations, and inadequate sampling of the conformational space ^8– 12^. Consensus based methods are based on the principle that structural features that are frequently observed in a population of structures are more likely to be present in the native structure ^13– 16^. These clustering methods outperform other MQAP methods ^14^ and are “very useful for structural meta-predictors ^17^”. However, they are prone to be computationally intensive due structure-to-structure comparison of all models ^16^, and are of limited use when the number of possible structures is small ^18^. Knowledge based methods proceed by deriving an empirical potential (also known as statistical potential) from the frequency of residue contacts in the known structures of native proteins ^19, 20^. For a system in thermodynamic equilibrium, statistical physics hypothesizes that the accessible states are populated with a frequency which depends on the free energy of the state and is given by the Boltzmann distribution. The Boltzmann hypothesis states that if the database of known native protein structures is assumed to be a statistical system in thermodynamic equilibrium, specific structural features would be populated based on the free energy of the protein conformational state. Applying a converse logic, Sippl reasoned that the frequencies of occurrence of structural features such as interatomic distances in the database of known protein structures could be used to assign a free energy (potential of mean force) for a given protein conformation ^21, 22^. Furthermore, this statistical potential can be used to discriminate the native structure ^23– 27^. The proper characterization of the reference state is a critical aspect in applying statistical potentials ^23^. In spite of their popularity, the application of such empirical energy functions to predict and assess protein structures are vigorously debated ^28, 29^. Many MQAP programs perform better when multiple statistical metrics are combined ^30– 33^. The paramount importance of obtaining high quality protein structures from sequences using *in silico* methods can be estimated by the effort invested by researchers every two years ^34^ to evaluate both structure prediction tools ^35^ and MQAPs ^17, 34, 36^.

Here, we propose a novel statistical potential to assess the quality of protein structures based on the electrostatic potential difference (EPD) of C *β* atoms in consecutive residues - EPD profile of sidechain atoms used in assessment of protein structures (ESCAPIST). Previously, we have established that the EPD is conserved in cognate pairs of active site residues in proteins with the same function ^37– 40^. The ability of finite difference methods to quickly obtain consistent electrostatic properties from peptide structures provides an invaluable tool for investigating other innate properties of protein structures ^41^. We plot the EPD profiles for different atom types (C *α* atoms, C *β* atoms and the C-N bond) in consecutive residues from a set of non-homologous protein structures obtained from the PISCES database ( http://dunbrack.fccc.edu/PISCES.php) ^42^. We proceed to show that the EPD between C *β* atoms in consecutive residues can be used to generate a scoring function that assesses the quality of protein structures. This EPD scoring function is then applied to standard decoy sets from the Decoys ‘R’ Us database ( http://dd.compbio.washington.edu) to establish the validity of our method ^43^.

## Results

### Electrostatic potential difference (EPD) based discrimination

To extract feature values we chose a set of 1000 proteins from the PISCES database with percentage identity cutoff of 20%, resolution cutoff of 1.6 Å and a R-factor cutoff of 0.25 (SI Table 1).

**Table 1.** Electrostatic potential differences (EPD) for consecutive residue pairs for C *α* atoms for residue pairs that include proline. While these pairs have for a low standard deviation (SD) like all other pairs, the absolute value of their mean is different (higher) than any pair that does not include a proline. This also highlights the unique nature of proline in protein structures.

Pair	Mean EPD	SD	Number of samples
AP	-167.3	28.6	328
CP	-153	30.3	45
DP	-184.5	29.5	290
EP	-176.6	27.3	346
FP	-160.4	25.3	173
GP	-165.3	29.2	339
HP	-162.7	34.6	92
IP	-161.9	27.2	175
KP	-156.6	29.6	203
LP	-165.2	28.3	323
MP	-161.3	29.5	70
NP	-159.6	27.6	168
PQ	168.5	26.1	131
PR	156.2	31.5	184
PS	172.3	25.9	269
PT	170.8	27.6	218
PV	164.4	30.4	299
PW	158.5	29.7	70
PY	155.5	29	141

### Invariance of the EPD in the C-N peptide bond and between C *α* atoms of consecutive residues

**Figure 1.**
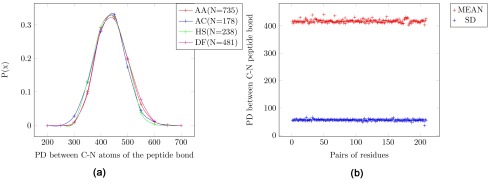
Electrostatic potential differences (PD) for the C-N peptide bond. AA: Alanine/Alanine, AC: Alanine/Cysteine, HS: Histidine/Serine and DF: Aspartic-acid/Phenylalanine. ( **a**) Probability distribution for four pairs of amino acids. ( **b**) Scatter plot for all pairs of amino acids. It can be seen that the mean and SD for all pairs of amino acids are the same. Further, the variance is large (SD=~50), indicating that this feature is not tightly constrained in peptide structures.

Adaptive Poisson-Boltzmann Solve (APBS) writes out the electrostatic potential in dimensionless units of kT/e where k is Boltzmann’s constant, T is the temperature in K and e is the charge of an electron. The units of EPD are same as that of the electrostatic potential. The EPD of the C-N peptide bond has a Gaussian distribution with mean = 420 EPD units and SD = 55 EPD units ( Figure 1). In the probability distribution for four pairs of amino acids the mean of all pairs of amino acids are the same ( Figure 1a). Figure 1b shows the scatter plot for the mean and standard deviation (SD). Thus, the amino acids are indistinguishable using the profile of the EPD of the C-N peptide bond across all protein structures since they have identical mean values and a large variance (SD= *~*50).

**Figure 2.**
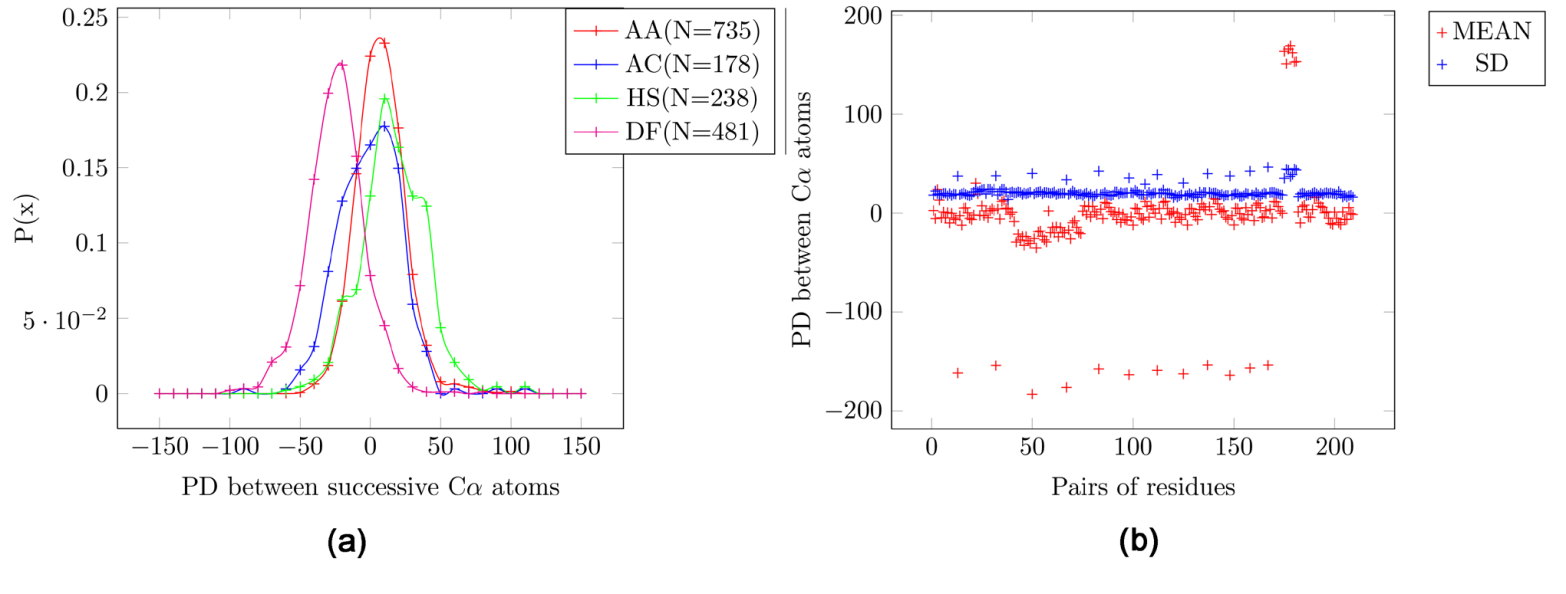
Electrostatic potential differences (PD) for consecutive residue pairs for C *α* atoms. A: Alanine/Alanine, AC: Alanine/Cysteine, HS: Histidine/Serine, DF: Aspartic-acid/Phenylalanine. ( **a**) Probability distribution for four pairs of amino acids. ( **b**) Scatter plot for all pairs of amino acids. It is seen that pairs of amino acids which include proline have a higher mean, although the magnitude of SD is the same.

The probability distribution for four pairs of amino acids for the EPD between the C *α* atoms of consecutive residues ( Figure 2a) have means that are slightly more varied than those for the C-N bond ( Figure 1a). In the scatter plot for the mean and SD of all pairs ( Figure 2b) the outliers are pairs that include proline, which have a higher mean, although the magnitude of SD is the same ( Table 1).

### Distinctive EPD between C *β* atoms of consecutive residues for certain amino acid pairs

**Figure 3.**
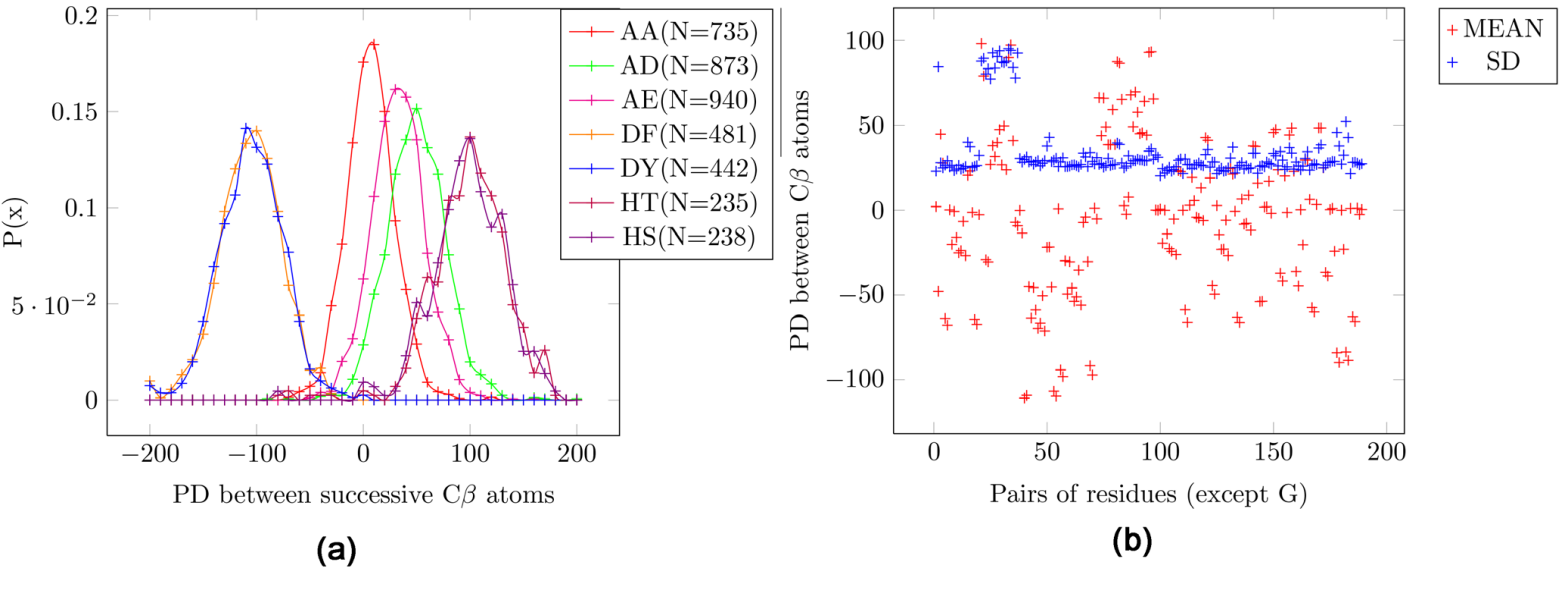
Electrostatic potential differences ( **PD**) for consecutive residue pairs for C *β* atoms. AA: Alanine/Alanine, AD: Alanine/Aspartic-acid, AE: Alanine/Glutamic-acid, DF: Aspartic-acid/Phenylalanine, DY - Aspartic-acid/Tyrosine, HT: Histidine/Threonine, HS: Histidine/Serine. ( **a**) Probability distribution for seven pairs of amino acids. ( **b**) Scatter plot for all pairs of amino acids. The pairs which include cysteine have a high standard deviation. It is seen that the mean is much more varied than the electrostatic potential difference (EPD) for C *α* and the C-N peptide bond.

In contrast to the results described above, the EPD between the C *β* atoms in consecutive residues in the peptide structure can be used to discriminate different amino acid pairs in the protein structure. The mean EPD of all amino acid pairs are much more varied ( Figure 3a). These pairs do not include glycine, which lacks a sidechain. In the scatter plot for the mean and SD, the outliers are pairs that include cysteine ( Figure 3b), which have a much higher SD (= *~*90) as compared to other pairs (SD= *~*35) ( Table 2), and thus cannot be used for discriminatory purposes.

**Table 2.** Electrostatic potential differences (EPD) for consecutive residue pairs for C *β* atoms for residue pairs that has one cysteine. These pairs have a random values for the mean and a high standard deviation (SD), with the exception of the pair ‘CC’ (not the disulfide bond) which has a low mean value and SD. Consequently, these values can not discriminate between pairs of amino acids.

Pair	Mean EPD	SD	Number of samples
AC	-53.7	86.9	178
CC	-7.1	30.4	36
CD	103.8	92.7	154
CE	96.8	94.7	121
CF	-21.4	84.2	85
CH	-12	93.3	97
CI	32.8	80.9	136
CK	50.2	93.9	131
CL	42.8	90.6	224
CM	61.9	100	39
CN	63.7	96.1	115
CP	24.9	88	45
CQ	66.7	92.1	95
CR	35.4	95.1	144
CS	106.3	98.1	184
CT	109.9	97.5	173
CV	54.9	90.7	183
CW	-0.2	85.9	43
CY	8.5	91.5	96

**Figure 4.**
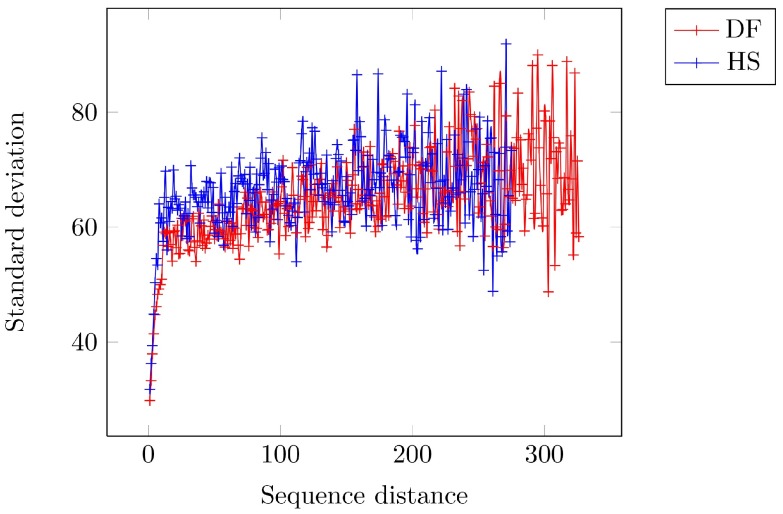
Standard deviation (SD) of the electrostatic potential difference between C *β* atoms increases with increasing sequence distance for amino acid pairs. Each sequence distance has at least 30 sample points. DF: Aspartic-acid/Phenylalanine, HS: Histidine/Serine. As expected, there is lesser correlation in the EPD values between the shown amino acid pairs ‘DF’ and ‘HS’ as the sequence distance between the residues increases. The SD for distance 1 (i.e. consecutive residues) is 29.8 EPD units and 31.8 EPD units for ‘DF’ and ‘HS’, respectively - and rises to around 60 EPD units with increasing sequence distance.

These values are used as a discriminator when choosing the native structure from a set of possible candidates ( Table 3). To establish the non-triviality of these values, we also show that the variance of the EPD between these pairs increases with increasing sequence distance. Thus, the EPD between the pairs ‘DF’ and ‘HS’ has lesser correlation as the sequence distance between them increases (sample size for each sequence distance is *>* 30) ( Figure 4). The SD for distance 1 (i.e. consecutive residues) is 29.8 EPD units and 31.8 EPD units for ‘DF’ and ‘HS’, respectively - and rises to around 60 EPD units with increasing sequence distance.

**Table 3.** Electrostatic potential differences (EPD) in a sample of consecutive residue pairs of C *β* atoms. These pairs are used for discriminating predicted structures in order to obtain the native structure. The complete set is available at https://github.com/sanchak/mqap.

Pair	Mean EPD	SD	Number of samples
DF	-108.9	29.5	481
DY	-107.4	30.7	442
DH	-105.2	33.5	242
DW	-104.1	27.7	209
EH	-98.5	28.5	200
EY	-96.5	28	378
EW	-94.2	29.8	184
SY	-93.5	27.5	403
EF	-93.1	27.6	439
TY	-93	28.6	384
TW	-90.8	28.7	144
SW	-89.2	27.7	169
FT	89.2	26.8	436
FS	92.3	28.4	453
HS	93.7	31.8	235
HT	95.1	31.5	235

### Validating using decoy sets

We obtained the score ( *PDScore*) of any given protein structure by comparing the electrostatics of the C *β* atoms based on Table 3. To benchmark model quality assessment programs, we used decoy sets from the Decoys ‘R’ Us database ^43^. We detail our results from some of these decoy sets. Each set has several structures that are supposed to be ranked worse than the native structure.

The misfold decoy set has *~*20 protein structures, each of which has a correct and an incorrect structure specified (three structures have two incorrect structures: we randomly chose the first) ^44^. The PDScore of these proteins were computed ( Table 4). Barring three structures (PDBids: 1CBH, 1FDX and 2SSI), the PDScore of the incorrect structure is higher than that of the correct structures.

**Table 4.** Misfold decoy set. This decoy set has ~20 protein structures - each of which has a correct and an incorrect structure specified. The PDBs are sorted based on the number of residues in the structure (NRes). Three of the structures (1CBH, 1FDX and 2SSI) have a lower PDScore for the incorrect structure.

PDB	NRes	Correct PDScore	Incorrect PDScore	Specificity
1CBH	36	18.7	12.6	0
1PPT	36	18	33.5	1
1FDX	54	33	30.9	0
5RXN	54	25.1	35	1
1SN3	65	20.7	30.3	1
2CI2	65	19.9	35.2	1
2CRO	65	26.7	43.4	1
1HIP	85	19.1	36.8	1
2B5C	85	22.1	34.3	1
2CDV	107	17.4	40.9	1
2SSI	107	22.6	20	0
1BP2	123	21	44.1	1
2PAZ	123	19.3	27.3	1
1P2P	124	28.6	29	1
1RN3	124	20.7	28.8	1
1LH1	153	18	26.2	1
2I1B	153	19	27.6	1
1REI	212	16.5	21.8	1
5PAD	212	18.8	32	1
1RHD	293	23.2	31.9	1
2CYP	293	21.2	35.8	1
2TMN	316	27.2	32.2	1
2TS1	317	21.3	28.2	1

The hg_structal set has about *~*30 proteins. Each protein has 30 structures (including the native structure). Table 5A shows specificity obtained for structures in this decoy set. The average specificity obtained for this decoy set is 0.91 ( Table 5A). The decoy set 4state_reduced has *~*600 structures for each of the seven proteins. We obtain an average specificity of 0.94 for this decoy set ( Table 5B). Similarly, for the ig_structal decoy set we obtain a specificity of 0.93 ( Supplementary Table 1).

**Table 5.** hg_structal and 4state_reduced decoy sets. The PDBs are sorted based on specificity. (A) The hg_structal decoy set has ~30 protein structures - each of which has 30 structures. The average specificity obtained for the set is 0.91. (B) The 4state_reduced decoy set has 7 protein structures - each of which has *~*600 structures. The average specificity obtained for the set is 0.94. (C) The fisa set has 4 protein structures - each of which has 500 structures. The electrostatic discriminator has low specificities in this case. We have previously demnostrated that this decoy set can be discriminated by a distance based criterion. It consists of physically nonviable structures, thus rendering an electrostatic analysis meaningless. NRes = number of residues, NStructures = number of structures in the decoy set.

	PDB	NRes	NStructures	Specificity
(A) hg_structal	2PGHA	141	30	0.2
	1MBS	153	30	0.5
	2DHBA	141	30	0.6
	1HDAB	145	30	0.9
	1MYT	146	30	0.9
	1HLM	158	30	0.9
	1HSY	153	30	0.9
	1MBA	146	30	0.9
	1MYGA	153	30	0.9
	1MYJA	153	30	0.9
	1ASH	147	30	1
	1BABB	146	30	1
	1COLA	197	30	1
	1CPCA	162	30	1
	1ECD	136	30	1
	1EMY	153	30	1
	1FLP	142	30	1
	1GDM	153	30	1
	1HBG	147	30	1
	1HBHA	142	30	1
	1HBHB	146	30	1
	1HDAA	141	30	1
	1HLB	157	30	1
	1ITHA	141	30	1
	1LHT	153	30	1
	2DHBB	146	30	1
	2LHB	149	30	1
	2PGHB	146	30	1
	4SDHA	145	30	1
(B) 4state_reduced	2CRO	65	675	0.8
	3ICB	75	654	0.9
	4RXN	54	677	0.9
	4PTI	118	688	1
	1CTF	131	631	1
	1R69	97	676	1
	1SN3	65	661	1
(C) fisa	4ICB	76	501	0
	1FC2	44	501	0.4
	1HDDC	57	501	0.1
	2CRO	65	501	0.7

## Discussion

The functional characterization of a protein from its sequence using *in silico* methods based on the ‘sequence to structure to function’ paradigm is contingent upon the availability of its 3D-structure. The rapidly developing field of next generation sequencing has exacerbated the bottleneck of obtaining structural data using crystallization techniques ^45^. This ever-widening gap has been filled by methods that predict structures from sequences ^46^, based either on features from databases of known structures ^2– 4^ or from first principles of atomic interactions ^5, 6^.

The various sources of error in protein structure prediction have been previously discussed in detail ^47^. An incorrect model of a protein structure will result in an inaccurate analysis of its properties ^48^. For example, continuum models ^49^ that compute potential differences and pK _a_ values from charge interactions in proteins ^50^ are sensitive to the spatial arrangement of the atoms in the structure. It must be pointed out that other detailed methods using quantum mechanical/molecular mechanical (QM/MM) techniques and and doing extensive conformational sampling have been able to determine the side chain pK _a_ values with high accuracy ^51^. Accurate structural information is indispensable for *in silico* methods that extract the electrostatic profile of atoms in the peptide structure ^41, 52^, and for other methods widely used in pharmacology for drug discovery ^53^. Model quality assessment programs (MQAP) that validate the accuracy of predicted structures are thus a critical aspect in the process of modeling a protein structure from its sequence. MQAPs can be classified as energy ^8– 12^, consensus ^13– 16^ or knowledge based (statistical potential) ^21– 27^. The state of the art methods for predicting structures ^35^ and MQAPs ^17, 34, 36^ are evaluated by researchers every two years.

Previously, we hypothesized and demonstrated that the electrostatic potential difference (EPD) in cognate pairs in the active site are conserved in proteins with the same functionality ^37, 40, 54^, even when evolution has converged to the same catalytic from completely different sequences ^55^. This similarity is observed in structures solved independently over many years and establishes the reliability of the APBS and PDB2PQR implementations ^41, 56^. We focused on unraveling other electrostatic features that are innate to protein structures. Here, we first demonstrate that the EPD between the C-N peptide bond and the C *α* atoms of consecutive residues are independent of the amino acid type. This is expected, since the distance between these atoms are almost invariant across all structures. The EPD of the C-N bond has a high variance, implying that the backbone accommodates relatively large variations while seeking energetically minimized structures.

The true source of the chemical and structural diversity in protein structures is the side chain atoms. Every amino acid, except glycine, has a C *β* atom that hosts a unique moiety of atoms. Although the reactive groups are different for amino acids, we show that this difference is encapsulated in the backbone C *β* atoms. We first show that different pairs of amino acids have significantly different mean EPD values in side chain C *β* atoms ( Figure 3), unlike the EPD of the C-N peptide bond ( Figure 1) or the EPD between consecutive C *α* atoms ( Figure 2). Further, the variance is much less than in the EPD of the C-N bond. These facts suggested that the EPD between C *β* atoms of consecutive residues in the peptide structure might act as a discriminator. Our hypothesis is based on the insightful Boltzmann law that lower energy conformations are disproportionately sampled, on the thermodynamic hypothesis ^57^ that the native structure has minimal energy, and the hypothesis that statistical derived features in known protein structures have a Gaussian distribution ^21^. We apply our discriminator to standard decoy sets from the Decoys ‘R’ Us database to establish the validity of the method ^43^.

Our work also highlights the unique properties of proline in the protein structure ^58^. This is evident from the different magnitude of EPD in consecutive C *α* atoms involving proline ( Table 1). Another noteworthy aspect is the large variation in EPD in consecutive C *β* atoms involving cysteine ( Table 2), demonstrating the unique role cysteine plays in providing flexibility to protein structures, a critical element in the evolution of complex organisms ^59^. The discrimination of C *β* atoms also provides a uniform basis for methods that require a single-atom representation of a residue. Such methods depend on a correct parameterization of the reactive atoms ^37^, a task further complicated by amino acids such as histidine which has two reactive atoms. For example, the EPD between the negatively charged E and D with respect to the aromatic phenylalanine is -108 and -93 EPD units, in spite of the difference in their reactive atom. Similarly, the EPD between alanine and the three aromatic amino acids (F, W and Y) are -67, -66 and -63 EPD units respectively.

We achieved an average specificity of 0.91, 0.94 and 0.93 on hg_structal, 4state_reduced and ig_structal decoy sets, respectively, taken from the Decoys ‘R’ Us database. We have previously demonstrated that the fisa decoy set can be discriminated by a distance based discriminator ^60^. ESCAPIST does not discriminate the native structure in this decoy set ( Table 5C). The physical implication of ESCAPIST results on the fisa decoy set, which has significant RMSD for backbone C *α* atoms, needs further elaboration. The input to a finite difference Poisson-Boltzmann (FDPB) analysis is a charge distribution that might be unfeasible due to energy functions other than electrostatics. For example, van der Waals force or the elastic bond length force components might prevent two atoms from being in close proximity. However, if such a physically impossible configuration were presented to a FDPB-based analysis tool, such as APBS ^41^, it would still generate an electrostatic potential landscape. Inferences based on this potential landscape would be incorrect due to its physical non-viability. Thus, before invoking the EPD constraints specified here, it is imperative that other spatial constraints that are rarely violated in structures are checked. Possibly for this same reason, MQAPs that combine many features in their scoring functions are superior. Moreover, it should be kept in mind that decoy sets, like most benchmarking sets, are prone to biases ^61^ and possible errors ^31^. In fact, the fisa decoy set has been shown to violate the van der Waals term ^61^. To summarize, we present a novel discriminating feature in protein structures based on the electrostatic properties of the side chain atoms. We validated this discrimination in several decoy sets taken from the Decoys ‘R’ Us database, and achieved high specificities in most decoy sets.

## Methods

Our proposed method has two phases. In the learning phase, we process multiple structures to extract the feature values - mean values of electrostatic potential difference (EPD) for each amino acid pair. These feature values are applied on query proteins to obtain a score (PDscore) that indicates the deviation of the feature values in the given structure from the ‘ideal’ values. Thus, a lower PDscore indicates a better candidate.

### Learning phase

Algorithm 1 shows the procedure LearnFeatures() that extracts the feature values from a set of proteins Φ *^LearningPhase^_proteins_* ( Equation 1). We ignore the first x= *IgnoreNTerm* and last y= *IgnoreCTerm* pairs of residues in the protein structure to exclude the terminals. For every consecutive pair of residues in the structure, we calculate the EPD (see below for method) between two specified atoms ( *atomP* and *atomQ*). Both *atomP* and *atomQ* are set to C *β* to obtain EPD values for C *β* atoms, while we set *atomP* to ‘C’ and *atomQ* to ‘N’ in order to obtain the C-N peptide bond EPD values. The mean (Mean learnt value - MLV) and standard deviation (SD) are computed for each amino acid type pair (AAType) in protein ( Equation 2), and the mean computed for the globals set of proteins (MLV( *AAType ^x^, AAType ^y^*)) for each pair of amino acid types ( Equation 3). Pairs whose EPD have a SD greater than a threshold value ( *sdThresh*, 50 by default) are ignored. Means for all significant pairs ( **ϕ*_pairMean_*) are noted to a file, which is the input to the quality assessment phase. The EPD between a pair of amino acid is order-independent - for example, the EPD statistics for the pair ‘AC’ (alanine-cysteine) includes the EPD of both ‘AC’ and ‘CA’ (with the sign reversed).

ΦproteinsLearning Phase={P, 1P2…PM}     (1)

$$MLV(AAType^{Res_{n}},AAType^{Res_{n+1}})Pi=\frac{\sum {}_{n=1+x}^{N-y-1}(EPD(Res_{n}(atomP),Res_{n+1}(atomQ)))}{\left(N-y-x-2\right)}\;\;\;\;\;(2)$$

$$MLV(AAType^{x},AAType^{y})=[\forall i=1\ldots M,and\;AAType^{x}and\;AAType^{y}](\frac{\sum {}_{n=1}^{M}(MLV(AAType^{x},AAType^{y})Pi)}{M}\;\;\;\;\;(3)$$

### Quality assessment phase

Algorithm 2 shows the function AssessEPDQuality() that generates the *PDscore* for a given protein from the template file generated by the learning phase. The set of proteins $\Phi_{\text{proteins}}^{\text{Assessment Phase}}$ consists of the native structure *P*_1_ and N-1 decoys structures ( Equation 4). Once again, barring x= *IgnoreNTerm* and y= *IgnoreCTerm* number of residues from the N and C terminals, the pairwise EPD for consecutive residues are computed. The absolute value of the difference of these values from their corresponding means, if they exist, in the template file is added to generate the absolute score ( Equation 5). This score is normalized with the number of residues that have been compared to obtain the final *PDscore*. In summary, the *PDscore* encapsulates the average distance of the EPD for the given atom pairs (it may be C *β*, C *α* or the C-N bond) of consecutive residues from their mean values. We hypothesize that in the native or a near native structure, the *PDscore* will be minimized for the EPD of C *β* atoms of consecutive residues, i.e. given a set of proteins *Φ _proteins_* consists of the native structure *P*_1_ and N-1 decoys structures, *P*_1_ will have the minimum PDscore ( Equation 6).

$$\Phi_{\text{proteins}}^{\text{Assessment Phase}}=\{P_{1},P_{2}\ldots P_{N}\}\;\;\;\;\;(4)$$

$$PDscore^{Pi}=\frac{\sum {}_{n=1+x}^{N-y-1}Abs(EPD(Res_{n}(atomP),Res_{n+1}(atomQ))-MLV(AAType^{Res_{n}},AAType^{Res_{n+1}}))}{\left(N-y-x-2\right)}\;\;\;\;\;(5)$$

$$\left[\forall i=2\ldots N](PDscore^{P1}<PDscore^{Pi})\;\;\;\;\;(6\right)$$

The top level procedure ESCAPIST() is shown in Algorithm 3. It invokes the function LearnFeatures() once, and applies the learnt values to assess the quality of structures based on the feature values obtained.

### Computing electrostatic potentials

Adaptive Poisson-Boltzmann Solver ^41^ (APBS) and the PDB2PQR package ^56^ package was used to calculate the potential difference between the reactive atoms of the corresponding proteins. The APBS parameters are set as follows - solute dielectric: 2, solvent dielectric: 78, solvent probe radius: 1.4 Å, Temperature: 298 K and 0 ionic strength. APBS writes out the electrostatic potential in dimensionless units of kT/e where k is Boltzmann’s constant, T is the temperature in K and e is the charge of an electron.

**Algorithm 1: LearnFeatures(): extract electrostatic potential difference (EPD) values from a given pair of amino acids**

**Input**: *ϕ _proteins_* = { *P*_1_ ··· *P _M_*} : *M* Proteins in the learning set

**Input**: *IgnoreNTerm*: Ignore this number of residues in the N Terminal

**Input**: *IgnoreCTerm*: Ignore this number of residues in the C Terminal

**Input**: *atomP*: Atom type in first residue

**Input**: *atomQ*: Atom type in second residue

**Input**: *sdThresh*: Threshfold for standard deviation of the EPD

**Output**: **ϕ*_pairMean_* = { *meanPDC*_1_ ··· *meanPDC _K_*}: Mean values of EPD between specified atoms

               of successive residues, there being K such significant pairs

**begin**

      /*K pairs of amino acid type (sorted: AC and CA are equivalent)*/

      /*Each set is initialize to be the null set*/

      
**ϕ*_pair_* = { **ϕ**_1_ *_PDC_ ··· *ϕ*_KPDC_*} :

      
**foreach** *P _i_ in *ϕ*_proteins_* **do**

           
*N* = NumberOfResidues( *P _i_*);

           
**for** *p ← IgnoreNTerm* **to** ( *N − IgnoreCTerm*) **do**

               
*q* = *p* + 1 ;

               /* Amino acid pairs are order independent */

               
*ResiduePairTypeString* = *ResidueTypeString*( *p*) + *ResidueTypeString*( *q*);

               
*ResiduePairTypeStringSorted* = *Sort*( *ResiduePairTypeString*;

               /* Reverse sign of potential difference accordingly */

               
*multfactor* = 1 ;

               
**if** *(ResiduePairTypeStringSorted != ResiduePairTypeString)* **then**

                  
*multfactor* = -1 ;

               
**end**

               
*PD* = PotentialDifference( *p, q, atomP, atomQ*) * *multfactor* ;

               /* Let the amino acid pair be the kth in the set **ϕ*_pair_* */

               InsertInSet( *PD*, **ϕ*_kPDC_*);

         
**end**

      
**end**

      /* Compute Mean and SD of each set - ignore pairs with SD greater than sdThresh*/

      
**ϕ*_pairMean_* = ∅;

      
**foreach** **ϕ*_ipair_ in *ϕ*_pair_* **do**

           ( *Mean _i_, SD _i_*) = MeanAndSD( **ϕ*_ipair_*);

           
**if** *(SD _i_ > sdThresh)* **then**

              Add( *Mean _i_, *ϕ*_pairMean_*);

           
**end**

      
**end**

      
**return** ( **ϕ*_pairMean_*);

**end**

**Algorithm 2: AssessEPDQuality()**

**Input**: *P*_1_ : Protein under consideration

**Input**: *IgnoreNTerm*: Ignore this number of residues in the N Terminal

**Input**: *IgnoreCTerm*: Ignore this number of residues in the C Terminal

**Input**: *atomP*: Atom type in first residue

**Input**: *atomQ*: Atom type in second residue

**Input**: *ϕ _pairMean_* = { *meanPDC*_1_ ··· *meanPDC _M_*}: Mean values of EPD between specified atoms of

           successive residues

**Output**: *PDscore*: Score indicating the normalized distance of the observed values from the (mean)

             learnt values from native structures

**begin**

      
*PDscore* = 0 ; *NumberCompared* = 0 ; *N* = NumberOfResidues( *P*_1_);

      
**for** *p ← IgnoreNTerm* **to** ( *N − IgnoreCT erm*) **do**

          
*q* = *p* + 1 ;

          /* Amino acid pairs are order independent */

          
*ResiduePairTypeString* = *ResidueTypeString*( *p*) + *ResidueTypeString*( *q*);

          
*ResiduePairTypeStringSorted* = *Sort*( *ResiduePairTypeString*;

          /* Reverse sign of potential difference accordingly */

         
*multfactor* = 1;

          
**if** *(ResiduePairTypeStringSorted != ResiduePairTypeString)* **then**

             
*multfactor* = -1 ;

          
**end**

          /* Let the amino acid pair be the kth in the set **ϕ*_pair_* */

          
*PD* = PotentialDifference( *p, q, atomP, atomQ*) * *multfactor* ;

          
**if** *(*∃ *meanPDC _k_)* **then**

             
*NumberCompared* = *NumberCompared* + 1 ;

             
*diff* = absolute( *PD − meanPDC _k_*);

             
*PDscore* = *PDscore* + *diff*;

           
**end**

      
**end**

      /* Normalize */

      
*PDscore* = *PDscore/NumberCompared*;

      
**return** ( *PDscore*);

**end**

**Algorithm 3: ESCAPIST(): Top level function**

**Input**: **ϕ*_proteins_*: Learning set

**Input**: *P*_1_: Protein to be scored

**Input**: *IgnoreNTerm*: Ignore this number of residues in the N Terminal

**Input**: *IgnoreCTerm*: Ignore this number of residues in the C Terminal

**Input**: *atomP*: Atom type in first residue

**Input**: *atomQ*: Atom type in second residue

**Input**: *sdThresh*: Threshfold for standard deviation of the EPD

**Output**: *PDscore*: Score indicating the normalized distance of the observed values from the (mean)

             learnt values from native structures

**begin**

      /* This is invoked once*/ **ϕ*_pairMean_* =

      LearnFeatures( **ϕ*_proteins_, IgnoreNTerm, IgnoreCTerm, atomP, atomQ, sdT hresh*);;

      
*PDscore* = *AssessEPDQuality*( *P*_1_ *, IgnoreNTerm, IgnoreCTerm, atomP, atomQ, *ϕ*_pairMean_*);

      
**return** ( *PDscore*);

**end**

## Figures and Tables

**Table S1.** Proteins from the PISCES database used for learning values. Set of 1000 proteins from the PISCES database with percentage identity cutoff of 20%, resolution cutoff of 1.6 Å, R-factor cutoff of 0.25, and a RDCC cutoff of 0.012 Å used to learn feature values.

1A62 1AH7 1AHO 1AIE 1ARB 1ATG 1B5E 1BGF 1BKR 1BX7 1C1K 1C4Q 1C5E 1C75 1C7K 1CC8 1CCW 1CO6 1CZP 1D4O 1D5T 1DJ0 1DK8 1DS1 1E29 1E2W 1E58 1E5K 1E7L 1EAQ 1EB6 1EGW 1ELK 1ELU 1ES9 1ET1 1EUV 1EUW 1EYH 1EZG 1F1E 1F46 1F86 1F8E 1F94 1FK5 1FSG 1FT5 1FX2 1G2R 1G5A 1G61 1G8A 1G8Q 1GCI 1GJ7 1GK9 1GKP 1GMU 1GMX 1GP0 1GPQ 1GQI 1GU2 1GUT 1GWM 1GXM 1GXU 1GY7 1H16 1H91 1HDO 1HX0 1HXI 1HYO 1HZ4 1I1W 1I27 1I2T 1I71 1IDP 1IFR 1IJY 1IO0 1IQZ 1IRQ 1ITX 1J0P 1J98 1JCD 1JEK 1JET 1JFB 1JHJ 1JI7 1JK3 1JKE 1JL1 1JO0 1JOV 1JX6 1JZ8 1K4I 1K5C 1K5N 1K7C 1K7J 1KAF 1KMT 1KNG 1KOE 1KQ6 1KQR 1KS8 1KT6 1KWF 1KYF 1L9L 1LC0 1LKK 1LLF 1LWB 1M15 1M1Q 1M2D 1M40 1M55 1M9Z 1MC2 1MF7 1MFW 1MJ5 1MKK 1MN8 1MSO 1MUN 1MUW 1MWQ 1MY7 1N08 1N8V 1NC5 1NF8 1NG6 1NKD 1NKG 1NKI 1NLQ 1NNF 1NQJ 1NTH 1NUY 1NWW 1NWZ 1NYC 1O06 1O7I 1O7J 1O82 1OAI 1OCY 1ODM 1OH0 1OK0 1OQJ 1OU8 1P1M 1P1X 1P6O 1P9G 1P9I 1PO5 1PQH 1PSR 1PZ4 1Q5Y 1Q6Z 1Q92 1QDD 1QGI 1QL0 1QLW 1QNR 1QQF 1QV9 1QW2 1QW9 1QZ0 1R5L 1R6J 1R6X 1R7J 1R9L 1RA0 1RFY 1RG8 1RKI 1ROC 1RTQ 1RTT 1RV9 1RW1 1RWH 1RYO 1RYQ 1S1D 1S29 1S9R 1SAU 1SFX 1SG4 1SJW 1SN9 1SQS 1SU8 1SVF 1SZH 1SZN 1T1D 1T2D 1T3Y 1T6F 1T6U 1T8K 1T92 1TCA 1TJP 1TP6 1TQG 1TT8 1TU9 1TUA 1TUK 1TZP 1U84 1UCR 1UCS 1UFY 1UGI 1UKF 1UOY 1UTE 1UTG 1UUY 1UWK 1UZ3 1V05 1V4P 1V5V 1V7Z 1V8H 1VBW 1VCC 1VK1 1VKK 1VL7 1VLY 1VMG 1VMH 1VQS 1VR7 1VYI 1VZM 1W0H 1W0N 1W23 1W4S 1W53 1W5R 1W66 1WB4 1WBH 1WC2 1WCW 1WDD 1WER 1WHI 1WLZ 1WN2 1WNA 1WNY 1WPA 1WS8 1WTJ 1WU9 1WVF 1WVQ 1WWI 1WY3 1X54 1X6I 1X6Z 1X9I 1XDN 1XG0 1XMK 1XOD 1XPP 1XQO 1XSZ 1XUB 1Y43 1Y6X 1Y8A 1Y9L 1YB3 1YD0 1YPY 1YQS 1YU0 1YXY 1Z2U 1Z3X 1Z6M 1ZCE 1ZGK 1ZHV 1ZHX 1ZL0 1ZMI 1ZMM 1ZR6 1ZV1 1ZZ1 256B 2A26 2A6Z 2ACF 2AEB 2AHF 2AIB 2AKZ 2AP3 2APJ 2ASB 2ASK 2AXW 2AYD 2B0A 2B4H 2B82 2B97 2B9D 2BAY 2BCM 2BDR 2BF9 2BKX 2BL8 2BT9 2BWF 2C0H 2C1V 2C2U 2C3V 2C4J 2C71 2C78 2C92 2CAR 2CB8 2CC6 2CCQ 2CCV 2CCW 2CG7 2CHH 2CI1 2CIU 2CIW 2CKK 2CPG 2CS7 2CVE 2CVI 2CWS 2CXN 2CXY 2CYJ 2CZL 2CZS 2D1S 2D3D 2D5W 2D8D 2DDX 2DE3 2DG5 2DKJ 2DKO 2DLB 2DPF 2DS5 2DSX 2DT8 2DVM 2DWU 2DXA 2E4T 2E6F 2E7Z 2EAB 2EB4 2EGV 2EHP 2EHZ 2ELC 2END 2ENG 2ERF 2F23 2F62 2FAO 2FB6 2FBA 2FCJ 2FCL 2FCW 2FHZ 2FJ8 2FNU 2FQ3 2FVV 2FVY 2FWH 2G30 2G3R 2G7O 2GB4 2GKE 2GKG 2GKP 2GOM 2GPI 2GS5 2GS8 2GUD 2GUI 2H1V 2H8E 2HDO 2HEU 2HOX 2HP0 2HX0 2I49 2I53 2IA7 2IAY 2IC6 2II2 2INW 2IP6 2J43 2J5Y 2J6B 2J8B 2J8K 2J9W 2JCB 2JEK 2JHF 2LIS 2MCM 2MHR 2NLS 2NLV 2NNU 2NQT 2NQW 2NR7 2NSZ 2NUH 2NXV 2O1Q 2O6N 2O9S 2OA2 2OB5 2OCT 2ODI 2OFC 2OFK 2OLM 2OOA 2OV0 2OVG 2OVJ 2OY9 2P02 2P17 2P2S 2P51 2P5K 2P6W 2P8G 2PHN 2PIE 2PMR 2PNE 2POF 2PR7 2PRV 2PTH 2PVB 2PW0 2Q5C 2Q9K 2QAP 2QE8 2QF4 2QGU 2QIP 2QJL 2QKV 2QML 2QNG 2QNK 2QNL 2QRL 2QSB 2QSK 2QUD 2R01 2R2Z 2R5O 2R9F 2RA9 2RBK 2RH2 2RKL 2UVO 2V03 2V1M 2V2P 2V33 2V3I 2V4X 2V8I 2V9V 2VBK 2VC8 2VHA 2VHK 2VK8 2VNG 2VOK 2VPB 2VQ2 2VQ4 2VZC 2W1J 2W2E 2W39 2W40 2W5Q 2W8X 2WAG 2WDS 2WE5 2WFI 2WH7 2WLV 2WOY 2WTP 2WZO 2X32 2X3M 2X46 2X49 2X4K 2X6W 2XBG 2XDW 2XET 2XOD 2XOL 2XOM 2XRH 2XTS 2XU3 2XW6 2XWV 2Y24 2Y6H 2Y71 2Y78 2YFO 2YLB 2YMV 2YV9 2Z5W 2Z6O 2Z6R 2Z72 2ZDP 2ZHJ 2ZHP 2ZNR 2ZUX 2ZXY 2ZZV 3A07 3A16 3ACX 3AGN 3AIA 3AJ7 3AKS 3ATV 3AWU 3B0G 3B0X 3B64 3B79 3B9W 3BB0 3BBB 3BEX 3BMZ 3BO6 3BOE 3BOG 3BPT 3BWH 3BWZ 3BXU 3BY4 3C5K 3C68 3C70 3C9A 3C9U 3CEC 3CIJ 3CIM 3CKM 3CLM 3COV 3CP7 3CT5 3CT6 3CU9 3CZX 3D2Q 3D32 3D3B 3D4E 3D9N 3DG6 3DHA 3DLC 3DSO 3E4G 3EER 3EOI 3EPW 3EUR 3EYE 3F1L 3F2Z 3F40 3F43 3F9X 3FDE 3FEA 3FIA 3FKE 3FMY 3FO3 3FR7 3G21 3G36 3G48 3G5T 3GA4 3GAE 3GBW 3GE3 3GHA 3GJY 3GKJ 3GKM 3GKR 3GMG 3GMX 3GO5 3GOC 3GVO 3GWI 3GZR 3H0N 3H4X 3H5J 3H8G 3HF5 3HKW 3HP4 3HWU 3HYQ 3I3Q 3I4G 3I4O 3I94 3IAR 3IE4 3IE7 3ILW 3IMK 3ISX 3IT3 3IV4 3IXL 3JS8 3JU4 3JYO 3JYZ 3K05 3K1U 3K67 3K6Y 3KB9 3KFF 3KKF 3KKG 3KLR 3KM5 3KQR 3KYZ 3LFK 3LHC 3LMZ 3LQB 3LWX 3M3P 3M6Z 3M9Q 3MAO 3MC3 3MD7 3MDQ 3MJO 3MMH 3MOL 3MQD 3MXN 3NBM 3NI0 3NO2 3NPD 3NYC 3O79 3O8M 3OCZ 3OD9 3ODV 3OIG 3ON9 3ORU 3OV9 3OXP 3OYV 3PD7 3PFT 3PLW 3PMC 3PMS 3POD 3PP5 3PSM 3PUC 3PVH 3Q39 3Q3Y 3Q46 3Q64 3Q7R 3QHB 3QPA 3QR7 3QU3 3QZM 3QZX 3R5T 3R5Z 3R87 3R9F 3RJU 3RKG 3RQ9 3RT2 3RX9 3RZN 3S2R 3S44 3S4E 3S6F 3S9X 3SEE 3SHG 3SQZ 3SU6 3SUK 3SWO 3T47 3T8J 3T92 3TEW 3TG0 3TK2 3TOW 3TT9 3TYT 3U65 3U99 3U9W 3UEJ 3UF7 3UI4 3ULJ 3ULT 3UP3 3URR 3US6 3UW3 3V39 3V46 3V68 3VEN 3VII 3VMK 3VQJ 3VWC 3VXJ 3VZX 3W0K 3ZQU 3ZSJ 3ZUC 3ZYP 3ZZP 4A02 4A4J 4A56 4A6Q 4A7U 4A9V 4AAZ 4ACJ 4AIW 4ANN 4AV5 4AXO 4AXY 4AYO 4B4U 4DD5 4DI9 4DQA 4DQJ 4DT5 4DUI 4DVC 4DYQ 4DZN 4E40 4EFP 4EGU 4EP4 4EQ8 4EQP 4ERR 4ERY 4ES8 4EU9 4F2F 4F54 4FCH 4FR9 4FS7 4FTF 4FZL 4G3O 4G41 4G7X 4G9P 4G9S 4GB5 4GC3 4GEI 4GJZ 4GVF 4GWB 4H14 4H4D 4H4J 4H4N 4H6Q 4H8E 4HBQ 4HE6 4HHR 4HNO 4HTG 4HU2 4HWM 4HY4 4I1K 4IGT 4IIL 4IUM 4J42 1CXQ 1D8W 1DD9 1DF4 1DFM 1DG6 1DY5 1EKQ 1F9V 1FCQ 1FIU 1FO8 1FYE 1G2Q 1G3P 1G6X 1GK7 1GPP 1GSO 1GVJ 1GVP 1H2C 1H99 1IN4 1IUQ 1J3A 1J77 1JL0 1JNI 1JUH 1K3X 1K4N 1K6X 1KGD 1KJQ 1KMV 1LMI 1LNI 1LQT 1LS1 1LU0 1LUC 1LYQ 1M0K 1M22 1M4L 1MG7 1MNN 1N3L 1N4W 1NLS 1NNX 1NXM 1NZJ 1O8B 1OD6 1OI0 1OI7 1PP0 1PZ7 1QG8 1QWY 1R5M 1RYL 1SX5 1T1U 1T9I 1TBF 1U7I 1UNQ 1USM 1V0W 1V2B 1V6P 1VD6 1VE4 1VJU 1VYK 1W5Q 1W7C 1WFB 1WHZ 1WPU 1WT6 1X91 1XJU 1XMT 1XYI

**Table S2.** ig_structal decoy set. The PDBs are sorted based on specificity: The ig_structal decoy set has ~61 protein structures - each of which has 61 structures. The average specificity obtained for the set is 0.97. NRes =: number of residues, NStructures =: number of structures in the decoy set.

PDB	NRes	NStructures	Specificity
1FPT	11	61	0
1IKF	233	61	0.5
1IGM	115	61	0.9
1ACY	211	61	1
1BAF	214	61	1
1BBD	219	61	1
1BBJ	211	61	1
1DBB	211	61	1
1DFB	212	61	1
1DVF	107	61	1
1EAP	213	61	1
1FAI	214	61	1
1FBI	214	61	1
1FGV	107	61	1
1FIG	214	61	1
1FLR	219	61	1
1FOR	210	61	1
1FRG	217	61	1
1FVC	109	61	1
1FVD	214	61	1
1GAF	214	61	1
1GGI	211	61	1
1GIG	210	61	1
1HIL	211	61	1
1HKL	214	61	1
1IAI	214	61	1
1IBG	213	61	1
1IGC	213	61	1
1IGF	214	61	1
1IGI	213	61	1
1IND	211	61	1
1JEL	230	61	1
1JHL	108	61	1
1KEM	217	61	1
1MAM	214	61	1
1MCP	220	61	1
1MFA	113	61	1
1MLB	214	61	1
1MRD	211	61	1
1NBV	219	61	1
1NCB	386	61	1
1NGQ	211	61	1
1NMB	385	61	1
1NSN	213	61	1
1OPG	214	61	1
1PLG	215	61	1
1RMF	219	61	1
1TET	211	61	1
1UCB	211	61	1
1VFA	108	61	1
1VGE	214	61	1
1YUH	211	61	1
2CGR	219	61	1
2FB4	214	61	1
2FBJ	213	61	1
2GFB	214	61	1
3HFL	223	61	1
3HFM	214	61	1
6FAB	214	61	1
7FAB	204	61	1
8FAB	206	61	1
