# Supplementary material for: The electrostatic profile of consecutive Cβ atoms applied to protein structure quality assessment
Source: F1000Res. 2014 Sep 16;2:243. Originally published 2013 Nov 13. [Version 3] doi: 10.12688/f1000research.2-243.v3 (PMC4257144; doi:10.12688/f1000research.2-243.v3)
Supplement: Peer review history

# Peer review: Referee response for version 2

**DOI:** 10.5256/f1000research.3039.r5376

**Published:** 2014-07-18

**Authors:** Patricia C Weber

**Affiliations:** 1 Imiplex LLC, Bristol, PA, USA

The quality of protein structure models is assessed by the geometry of adjacent C beta atoms. The approach successfully distinguishes properly folded proteins in most cases. It adds a new way to assess protein models and could be included in the protein model assessment toolbox.

Just a few comments:

a) Table 4 lists three of 20 structures where the incorrect one has a lower score. A few comments about structural features of those examples that lead unexpected scores would be useful.

b) It might be preferable to note in the title that the method is applied to computational models of protein structure as a way to distinguish the manuscript from those that deal with quality assessment of experimentally determined structures.

c) I did not test the available source code.

I have read this submission. I believe that I have an appropriate level of expertise to confirm that it is of an acceptable scientific standard.

# Response

**Published:** 2014-09-15

**Authors:** Sandeep Chakraborty

**Affiliations:** Tata Institute of Fundamental Research, India

We would like to thank you for taking the time and reviewing our paper, and deeply appreciate your positive comments. We do not have the expertise to comment on the structural characteristics that lead to the 3 negative results in Table 4 - these require insights into crystal structures that we do not possess at the present time. Also, we believe that this method could be applied to any structure - computational or experimentally obtained - and thus are leaving the title unchanged.

# Peer review: Referee response for version 2

**DOI:** 10.5256/f1000research.3039.r4062

**Published:** 2014-03-12

**Authors:** Shina Caroline Lynn Kamerlin

**Affiliations:** 1 Computational and Systems Biology, Department of Cell and Molecular Biology, Uppsala University, Uppsala, Sweden

This is an interesting idea that uses the physical (electrostatic) properties of amino acid side chains in order to predict secondary structure from sequence, and thus assess (and rank) the quality of protein structures. The manuscript is well-written, and the authors provide comprehensive information to allow others to follow the study-design and methodology. The focus on electrostatics is important as this has been repeatedly shown by rigorous theoretical studies (work by Warshel and others) to be the primary driving force in determining protein function and most likely folding stability as well (whether directly or indirectly). As the specific methodology the authors use is slightly further from my area of expertise I cannot directly comment on this, however, importantly the source code has been made Open Access and freely available through Github.

My only comment is on the second paragraph of the Discussion, which comments on the pitfalls when using an incorrect model of a protein structure, particularly when trying to calculate pK _a_s using continuum models which are dependent on the initial conformation. While the authors highlight a very important challenge, I would like to point out that it can to some extent be resolved by extensive conformational sampling (particularly the pK _a_ problem, as the pK _a_ is an average property over all possible protein conformations), which we discussed at length in a review a few years ago ( Kamerlin *et al.*, 2009). Otherwise this is a nice manuscript and a valuable contribution to the literature.

I have read this submission. I believe that I have an appropriate level of expertise to confirm that it is of an acceptable scientific standard.
